# Effects of Delta-9 Tetrahydrocannabinol (THC) on Oocyte Competence and Early Embryonic Development

**DOI:** 10.3389/ftox.2021.647918

**Published:** 2021-03-23

**Authors:** Megan J. Misner, Afton Taborek, Jaustin Dufour, Lea Sharifi, Jibran Y. Khokhar, Laura A. Favetta

**Affiliations:** Department of Biomedical Sciences, University of Guelph, Guelph, ON, Canada

**Keywords:** fertility, oocyte, embryo, cannabis, THC

## Abstract

Recent changes in legal status and public perception of cannabis have contributed to an increase use amongst women of reproductive age. Concurrently, there is inadequate evidence-based knowledge to guide clinical practice regarding cannabis and its effects on fertility and early embryonic development. This study aimed to evaluate the effects of the primary psychoactive component of cannabis, delta-9 tetrahydrocannabinol (THC), during oocyte maturation, and its impact on the developing embryo. Bovine oocytes were matured *in vitro* for 24 h under clinically relevant doses of THC mimicking plasma levels achieved after therapeutic (0.032 μM) and recreational (0.32 and 3.2 μM) cannabis use. THC-treated oocytes were assessed for development and quality parameters at both the oocyte and embryo level. Characteristics of oocytes treated with cannabinoid receptor antagonists were also assessed. Oocytes treated with 0.32 and 3.2 μM THC, were significantly less likely to reach metaphase II (*p* < 0.01) and consequently had lower cleavage rates at day 2 post-fertilization (*p* < 0.0001). Treatment with cannabinoid receptor antagonists restored this effect (*p* < 0.05). Oocytes that did reach MII showed no differences in spindle morphology. Oocytes treated with 0.032 μM THC had significantly lower connexin mRNA (*p* < 0.05) (correlated with decreased quality), but this was not confirmed at the protein level. At the blastocyst stage there were no significant differences in developmental rates or the proportion of trophectoderm to inner cell mass cells between the control and treatment groups. These blastocysts, however, displayed an increased level of apoptosis in the 0.32 and 3.2 μM groups (*p* < 0.0001). Our findings suggest a possible disruptive effect of cannabis on oocyte maturation and early embryonic development.

## Introduction

Oocyte maturation ensures the oocyte has all the needed material to successfully undergo fertilization and progress through the early stages of embryonic development (De Felici et al., 2005; Sánchez and Smitz, 2012). In mammals, this initial development involves a number of tightly regulated signaling and molecular pathways, many of which are not fully elucidated (Park et al., 2004; Wang et al., 2006; Correa et al., 2016; Cecconi et al., 2020). This process as well as reproduction as a whole, is known to be susceptible to manipulation by exogenous environmental and lifestyle factors (De Angelis et al., 2020). With the decline of human fertility worldwide, such modifiable factors, including diet, exercise, alcohol use and smoking, have been extensively studied to better characterize the effect they play on fertility outcomes (Anderson et al., 2010; Van Heertum and Rossi, 2017; De Angelis et al., 2020). While a lot of work has been published in this regard, there still remain substances in which the available literature is inconclusive. Cannabis falls into this realm of understudied, but readily available compounds, which is especially notable with its recent legalization in many countries (Chavarro, 2018; Ilnitsky and Van Uum, 2019; De Angelis et al., 2020).

The study of the human reproductive system is challenging with respect to ethical standards, underscoring the need for establishing appropriate translational models; with several animal models (e.g., mouse, pig, cow) utilized over the years (Ménezo and Hérubel, 2002; Santos et al., 2014). Analysis of these species has determined the bovine reproductive model to be very translationally valid, due to the single ovulatory nature of the bovine species as well as the similarity in size of both male and female gametes to humans (Ménezo and Hérubel, 2002; Campbell et al., 2003; Luciano et al., 2010; Beker van Woudenberg et al., 2012; Santos et al., 2014). Furthermore, developmental parameters are most similar between cow and human, including oocyte maturation dynamics (Ménezo and Hérubel, 2002; Campbell et al., 2003; Santos et al., 2014) supporting the use of the bovine model in our study.

Cannabis use has been steadily increasing, most notably in the 15–35 age group, in which human fertility should be at its highest (National Institute on Drug Abuse, 2018; Ilnitsky and Van Uum, 2019; Statistics Canada, 2020). In the last quarter of 2019, 24 and 26.9% of Canadian respondents ages 15–24 and 25–34 years, respectively, reported cannabis use (Statistics Canada, 2020). A similar trend is seen in their American counterparts, with 22% of 18–25-year-old reporting regular cannabis use in 2017 (National Institute on Drug Abuse, 2018). At the same time, it has been reported that cannabis use has also been increasing during pregnancy (Corsi et al., 2019). Furthermore, a study of Canadian prenatal patients found that 92% used cannabis to control hyperemesis gravidarum and reported it to be very effective, thereby strengthening the need for more research on the effects of cannabis in early pregnancy (Westfall et al., 2006).

The cannabis plant, *Cannabis sativa* is composed of two main compounds, cannabidiol and delta-9 tetrahydrocannabinol (THC), with the latter highly studied due to its ability to elicit psychoactive effects (Mechoulam and Gaoni, 1965; Huestis et al., 1992; Childers and Breivogel, 1998; Cecconi et al., 2020). Furthermore, selective breeding of the cannabis plant has also led to an overall increase in THC content, thereby increasing the potency of cannabis preparations (El Sohly et al., 2016).

Upon absorption by tissues, THC is able to bind to receptors that belong to the body's pro-homeostatic endocannabinoid system (ECS) (Lemberger et al., 1971; Maykut, 1985; Dalton et al., 2009; Maccarrone et al., 2010, 2015; Pertwee et al., 2010; Di Blasio et al., 2013; Walker et al., 2019). Anandamide (AEA) and 2-arachidonoylglycerol (2-AG) are fatty acid derived eCBs known to act as agonists at the 2 main cannabinoid receptors: cannabinoid receptor 1 (CB1) and cannabinoid receptor 2 (CB2) (Huestis et al., 1992; Maccarrone et al., 2010, 2015; Pertwee et al., 2010; Di Blasio et al., 2013; Fonseca et al., 2013; Walker et al., 2019; Cecconi et al., 2020). The CB1 receptor, which was first localized in the nervous tissue (Joshi and Onaivi, 2019; Cecconi et al., 2020), has now been identified throughout the body, including the reproductive system, and inclusive of the ovaries and oocytes (El-Talatini et al., 2009; López-Cardona et al., 2016; Joshi and Onaivi, 2019). Furthermore, studies in mice have found the levels of uterine anandamide and blastocyst CB1 to be down-regulated with uterine receptivity (Schmid et al., 1997), with CB1 levels remaining high in non-receptive uteri. A strict regulation of the endocannabinoid signaling is necessary for synchronizing uterus' receptivity and embryo implantation (Paria et al., 2001). In this regard, THC has been characterized as an exocannabinoid for its ability to mimic the effects of the eCBs and activate both CB1 and CB2 receptors (Lemberger et al., 1971; Maykut, 1985; Dalton et al., 2009; Pertwee et al., 2010).

Upon binding to the ECS receptors, both the eCBs and THC elicit a wide array of signaling pathways via CB1 and CB2 (Howlett et al., 2002; Karasu et al., 2011; Cecconi et al., 2020), culminating in loss in intracellular cyclic adenosine monophosphate (cAMP), changes to gene transcription and promotion toward an apoptotic state (Guindon and Hohmann, 2011; Fonseca et al., 2013; Cecconi et al., 2020). Previous studies have suggested that these pathways coordinated by the ECS may be involved in the regulation and control of oocyte maturation by the ECS (Schuel et al., 2002; Lazzarin et al., 2004; Taylor et al., 2007; Battista et al., 2008; Karasu et al., 2011; Cecconi et al., 2020). The exact role of the eCBs (AEA and 2-AG) during oocyte maturation, however, as well as the molecular mechanisms responsible for this process have not been fully elucidated. Studies using samples collected from *in vitro* fertilization patients have also noted a positive correlation between concentration of AEA in follicular fluid and increasing oocyte maturity (Lazzarin et al., 2004; El-Talatini et al., 2009). Cannabis use in females has been linked to menstrual cycle disturbances, decreased follicle maturation and fewer embryos transferred during assisted reproduction procedures (Mueller et al., 1990; Klonoff-Cohen et al., 2006).

In males, cannabis use has been associated with oligospermia (low sperm count), decreased sperm motility and abnormal morphology of spermatozoa (Whan et al., 2006; Gundersen et al., 2015). The regulation of the endocannabinoid system has been shown to be necessary for the preservation of normal sperm function and male fertility, as seminal plasma AEA levels are lower in men with asthenozoospermia or oligoasthenoteratozoospermia compared with normozoospermic men, suggesting an involvement of eCBs in the reproductive system (Amoako et al., 2013).

A study conducted by Whan and colleagues utilized physiologically relevant doses of cannabis by separating cannabis use into two main purposes: therapeutic and recreational. Therapeutic use comes in the form of the FDA approved drug Marinol, which is used for its anti-emetic and appetite stimulant properties (FDA, 2004). Upon use at the recommended dosage, the levels of THC reach their peak plasma concentrations of 0.025 μM (±0.015) at 1.5 h (FDA, 2004). Recreational dosage was cited as both a low and high concentration due to the variability of use including chronicity and route of absorption, with plasma concentrations between 0.32 and 4.8 μM (Whan et al., 2006).

The area of endocannabinoid research in reproduction is rapidly advancing. As of 2020, there was no conclusive evidence linking cannabis doses with concentrations of THC within follicular fluid. Therefore, plasma levels as noted by Whan et al. (2006) were utilized to provide results for a wide range of concentrations.

The present study aimed to provide evidence for the effect of THC on oocyte maturation and the subsequent early embryonic development. Through analysis of various *in vitro* developmental parameters, embryo quality, and downstream targets of ECS activation, further indications into the effect of THC on fertility and early embryonic development have been identified. Maturation, cleavage and blastocysts rates were assessed as a mean to measure embryo developmental capability, while apoptosis and differential nuclear staining at the blastocyst level have been investigated to determine embryo quality. Connexin levels were used as indicator of oocyte competency, as it has been proven to be highly correlated to fertilization potential of oocytes and embryo quality (Wang et al., 2012). The results obtained here provide a first layer of evidence on the effects of cannabis at the oocyte and embryo levels that are needed to further establish clinical guidelines in the near future.

## Materials and Methods

### Experimental Design

Bovine cumulus oocyte complexes (COCs) were matured *in vitro* in one of five treatment groups to compare the effects of multiple doses of THC representing plasma levels achieved after therapeutic and recreational cannabis use (Huestis et al., 1992; Howlett et al., 2002; FDA, 2004; Whan et al., 2006). The groups were as follows: control, vehicle control (3.2 μM of 1:1:18 ethanol:Tween:saline), therapeutic/low (THC) (0.032 μM THC), recreational/mid (THC) (0.32 μM) and recreational/high (THC) (3.2 μM). At present, there is no literature outlining the levels of THC within follicular fluid after cannabis use, and thus a multi-dilution system was utilized based on plasma THC levels.

### Chemicals

Unless otherwise noted, chemicals and reagents used in this study were obtained from Sigma Aldrich, Oakville, Canada.

### *In vitro* Maturation of Cumulus-Oocyte Complexes

Bovine (*Bos taurus*) ovaries were collected from the local abattoir (Cargill Meat Solutions, Guelph, ON, Canada) and follicles were aspirated and collected into a vacutainer tube containing 1 mL of oocyte collection media: 1M HEPES 52—buffered Ham's F-10 media supplemented with 2% steer serum (Cansera International Inc, ON, Canada), heparin (2 IU/mL), sodium bicarbonate and 1% penicillin/streptomycin (Gibco, ON, Canada). High quality COCs with a dark, homogenous oocyte and well-defined cumulus cloud were randomly distributed into one of five treatment groups. The COCs were matured in serum *in vitro* maturation media (S-IVM) comprised of HEPES buffered TCM199 maturation media supplemented with 2% steer serum, sodium pyruvate, 1 μg/mL luteinizing hormone (LH) (NIH, Washington, DC, USA) 0.5 μg/mL follicle stimulating hormone (FSH) (Follitropin V; Vetoquinol, QC, Canada), 1 μg/mL estradiol and 10% fetal bovine serum (FBS) (Gibco). The groups were distinguished by the addition of either vehicle (1:1:18 ethanol:tween 20:saline) or THC (Dronabinol; Toronto Research Chemicals, ON, Canada). COCs were matured in the micro-drops of group-specific media for 22–24 h at 38.5°C in 5% CO_2_.

For the antagonist studies, the five treatment groups were as follows: control, vehicle, high (THC) (3.2 μM), high (THC) + 3.2 μM CB1 antagonist (SR141716; NIMH Drug Repository Program) and high (THC) + 3.2 μM CB2 antagonist (SR144528; NIMH Drug Repository Program). All other parameters were kept consistent.

### *In vitro* Fertilization (IVF)

*Bos taurus* semen (Semex, Guelph, ON, Canada) was collected from liquid nitrogen storage and thawed in a water bath at 36–38°C for 30 s. Sperm was prepared using the swim-up technique in HEPES sperm TALP supplemented with 15% bovine serum albumin (BSA) in an incubator set at 38.5°C and 5% CO_2_ for 45 min. Mature COCs were washed twice in HEPES + 15% BSA and then twice in BSA-supplemented IVF Tyrode albumin lactate pyruvate (TALP). At the culmination of swim-up, the upper layer of each swim-up tube was aspirated and centrifuged at 200xg for 7 min. The upper layer was discarded, and the sperm pellet was reconstituted in IVF TALP + BSA. Sperm motility was analyzed, and washed COCs were fertilized at a concentration of 1 × 10^6^ sperm cells/mL/drop. The HEPES + 15%BSA micro-drops with the gametes were placed into the incubator at 38.5°C and 5% CO_2_ for 18 h.

### *In vitro* Culture (IVC)/Embryo Production

At 18 h post-fertilization, the remaining cumulus cells were stripped using mechanical disruption. Presumptive zygotes were washed in HEPES + BSA followed by synthetic oviductal fluid (SOF) media supplemented with freshly prepared sodium pyruvate, gentamicin, essential amino acids, non-essential amino acids, 15% BSA and 2% FBS. Washed presumptive zygotes were placed in micro-drops of SOF covered in mineral oil and set in the incubator at 38.5°C and 5% O_2_ for culture to the blastocyst stage. Cleavage rate was recorded on day 2 post-fertilization and blastocyst rate at day 8. Data from 11 replicates of at least 60 oocytes/group were analyzed for cleavage rate, totalling 836 oocytes/group. For blastocyst rate, 7 replicates of at least 60 oocytes/group were used, totalling 507 oocytes/group.

### Oocyte Maturation Rates

Following 24 h of IVM, COCs were collected and placed in warmed hyaluronidase from bovine testes. The denuded oocytes were washed three times in sterile phosphate buffered saline (PBS) with 0.01% polyvinyl alcohol (PVA). During this process, the oocytes with visible polar bodies (and therefore assumed to have matured to the MII stage) were separated from the immature. The oocyte maturation rate was calculated by using the ratio of total oocytes with a polar body over the total number of oocytes in that group. Three replicates of 50+ oocytes/group were used, with 164 oocytes/group analyzed in total.

### Immunocytochemistry for Oocyte Meiotic Spindles

Denuded oocytes that had progressed to the MII stage (oocyte maturation rate protocol) were collected and fixed in 4% paraformaldehyde (PFA) for 20 min at room temperature followed by 1% PFA for storage at 4°C. Briefly, fixed oocytes were removed from the 1% PFA and moved through two washes of tris-buffered saline (pH 7.6) plus 0.1% Tween 20 (TBST). Oocytes were transferred into 0.5% Triton X-100 for 90 min, followed by blocking in 1% goat serum (Millipore Canada) for 1 h. Oocytes were then placed into the primary antibody, monoclonal alpha-tubulin (Sigma Aldrich, Cat# T5168) at a 1:500 dilution (in 0.5% goat serum in TBST) overnight at 4°C. The negative control was incubated in 1% goat serum only. Subsequently, all gametes underwent a total of four TBST washes and incubated with goat anti-mouse IgG (H+L), Superclonal™ Recombinant Secondary Antibody, Alexa Fluor 488 (Thermo Fisher Scientific, Cat# A28175) at a 1:500 dilution in 0.5% goat serum in TBST. All oocytes were incubated with the secondary antibody overnight, in the dark at 4°C. The oocytes were then washed three times in TBST for 30 min each, placed in PBS/PVA then mounted on slides using Vectashield with DAPI and covered with a coverslip. Oocytes were imaged with an Olympus FV120 confocal microscope at 40 × objective in air and 60 × objective in oil magnification using the Fluoview software. Seventeen replicates of at least 5 oocytes/group were stained for a total of 70–83 oocytes examined/group.

### Differential Staining and Total Nuclei Count in Blastocysts

Blastocysts from each group were washed three times in PBS/PVA and incubated in RNase solution (Fisher Scientific) (at 100 mg/mL in PBS) for 60 min at 38.5°C. The blastocysts were then washed once in PBS/PVA and transferred into propidium iodide (PI) solution (0.4% PI in Triton X-100) for 30 s at room temperature. After three washes in PBS/PVA, the embryos were placed in Hoechst stain (10 μg/mL in 4% PFA) for 15 min at room temperature. The blastocysts were again washed in PBS/PVA and transferred onto slides containing Vectashield antifade mounting medium and covered with a coverslip. Blastocysts were imaged using a Leica CTR5500B fluorescent microscope at the 20 × objective using the OpenLab software. Counts of cells in the trophectoderm (TE) and inner cell mass (ICM) were carried out both manually and via ImageJ software, however, only manual counts were used for statistical analysis. A sample size between 4 and 14 biological replicates (blastocysts) were examined in each treatment group. The total number of cells was tallied and added to the Hoechst-stained blastocysts used for TUNEL staining to analyze the total nuclei count.

### RNA Extraction and Reverse Transcription

Total RNA was extracted from pools of 15 COCs at 22-h maturation and 5 blastocysts at day 8 post-fertilization using the Qiagen RNAeasy Plus Micro kit following the manufacturer's protocol. RNA extracted from 15 COCs was then quantified using a Nanodrop 2000c spectrophotometer (Thermo Scientific) and diluted with RNase-free water to a final concentration of 12.5 ng/μL RNA. For blastocysts, each replicate was calibrated by using the same number of blastocysts. Reverse transcription was carried out using QuantaBio qScript cDNA SuperMix (VWR) and a T100 Thermal Cycler (Bio-Rad). The protocol for this step was as follows: 5 min at 25°C, 30 min at 42°C, 5 min at 85°C and concluding with a return to 4°C.

### Reference Gene Selection

Appropriate reference genes stable during THC treatment were first selected using the reference gene software, geNorm and quantitative PCR (qPCR), following the MIQE guidelines (Bustin et al., 2009). Four candidate genes were considered: *tyrosine 3-monooxygenase/tryptophan 5- monooxygenase activation protein zeta* (YWHAZ) (Sharma, 2016), *glyceraldehyde 3- phosphate dehydrogenase* (GAPDH) (Ferris et al., 2016), *peptidylprolyl isomerase A* (PPIA) (Ferris et al., 2016), and β*-actin* (ACTB) (Tscherner, 2017) (Table 1). GeNorm software identified the most stable housekeeping genes to utilize as an appropriate reference value. As such, YWHAZ and GAPDH were used as the reference genes to normalize ddPCR data to.

**Table 1:** mRNA primers for candidate genes.

**Gene reference name**	**Gene full name**	**Product size (bp)**	**GenBank accession #**	**Primer sequence—forward and reverse (5^**′**^-3^**′**^)**	**Efficiency (%)**	**Source**
YWHAZ	*Tyrosine 3-monooxygenase/tryptophan 5-monooxygenase activation protein zeta*	120	NM_174813.2	F: GCARCCCACAGACTATTTCC R: GCAAAGACAATGACAGACCA	100.30	Sharma, 2016
GAPDH	*Glyceraldehyde 3-phosphate dehydrogenase*	153	NM_001034034.2	F:TTCCTGGTACGACAATGAATTTG R: GGAGATGGGGCAGGACTC	99.50	Ferris et al., 2016
PPIA	*Peptidylprolyl isomerase A*	111	NM_178320.2	F: TCTTGTCCATGGCAAATGCTG R: TTTCACCTTGCCAAAGTACCAC	103.70	Ferris et al., 2016
ACTB	Beta-actin	186	NM_173979.3	F: CCTTCCTGGGCATGGAATCCT R:TCTTCATTGTGCTGGGTGCC	97.00	Tscherner, 2017

### Droplet Digital PCR (ddPCR)

ddPCR was used to quantify the expression of *connexin 37* (CX37) (Yuan et al., 2008) and *connexin 43* (CX43) (Tesfaye et al., 2007) in both COCs and blastocysts (Table 2) as well as the expression of *caspase-9* (cas-9) in blastocysts. For connexin expression, cDNA of 15 COCs was first diluted with RNase-free water to 1:5 for the CX37 target and to 1:50 for CX43 and the 2 reference genes, YWHAZ and GAPDH in order to achieve the optimal target range of the ddPCR instrument (between 1 and 120,000 copies/20μL). 9 biological replicates were carried out for CX37 and CX43 in COCs. For blastocysts, the cDNA of 5 blastocysts remained undiluted due to the overall low concentration and 5 biological replicates were conducted. EvaGreen ddPCR Supermix (Bio-Rad) was used following the manufacturer's protocol and the Bio-Rad QX200 ddPCR system. The PCR cycle was as follows: 5 min at 95°C to activate the EvaGreen enzyme, 50 cycles of denaturation at 95°C for 30 s, annealing at 58°C for 90 s and elongation at 53°C for 90 s, followed by 5 min at 4°C, 5 min at 90°C (for signal stabilization) and finishing off with 10 min at 4°C. All samples were run in technical duplicates including a negative control (RNase-free water) and a no template control (NTC). Results were normalized to the reference genes selected by geNorm: YWHAZ and GAPDH.

**Table 2:** mRNA primers used for ddPCR.

**Gene reference name**	**Gene full name**	**Product size (bp)**	**GenBank accession #**	**Primer sequence—forward and reverse (5^**′**^-3^**′**^)**	**Efficiency (%)**	**Source**
CX37	*Connexin 37/Gap junction protein alpha 4*	221	NM_001083738.1	F: GACTCATCTCCCTGGTGCTC R: GTTCTGCTCACTGGACGACA	97.00	Yuan et al., 2008
CX43	*Connexin 43/Gap junction protein alpha 1*	104	NM_174068	F: GTCTTCGAGGTGGCCTTCTTG R: AGTCCACCTGATGTGGGCAG	101.90	Tesfaye et al., 2007

Cas-9 expression was measured with the TaqMan probe system (Thermo Fisher Scientific). Undiluted cDNA of 5 blastocysts/treatment group was used and 4 biological replicates were conducted. Multiplexing of TaqMan probes was utilized with the cas-9 assay having a FAM label (Bio-Rad, Bt04282449) and YWHAZ being VIC labeled (Bio-Rad, Bt01122444). ddPCR was carried out using the manufacturer's protocol and ddPCR Supermix for Probes (Bio-Rad). The PCR cycle was as follows: enzyme activation for 10 min at 95°C, 45 cycles of denaturation for 30 s at 95°C then annealing/extension for 90 s at 60°C, followed by 10 min at 98°C and a subsequent 10 min at 4°C. Cas-9 expression was normalized to the reference gene, YWHAZ.

### Western Blotting

Quantification of CX37 and CX43 proteins in COCs was performed by western blotting. Groups of 40 frozen COCs were thawed and lysed using radioimmunoprecipitation assay (RIPA) buffer and protease inhibitors (Biotool). This was followed by four freeze-thaw cycles in liquid nitrogen and placement in a sonicator for 30 min to maximize protein extraction. After centrifugation, reducing buffer with β-mercaptoethanol was added to the pellet. Protein samples were heated to 90°C for 6 min for protein denaturation. Mouse brain and MCF7 cell lysate (Abcam, Cat# ab3871) were used as positive controls for CX43 and CX37, respectively, and GAPDH was blotted as the loading control. 8% polyacrylamide gels were prepared using Bio-Rad standard gel recipes. Proteins samples were loaded onto the gel in an Invitrogen wet transfer western blot apparatus for 2 h at 125V in running buffer, followed by transfer to a nitrocellulose membrane.

The CX43 primary antibody (Sigma-Aldrich, Cat# C6219) was used at a 1:8,000 dilution overnight and the secondary antibody, Anti- Rabbit IgG HRP-linked antibody (Cell Signaling Technology, Cat# 7074) was incubated at a dilution of 1:5,000 for 1 h. The blot was washed in TBST three times and incubated with UltraScence Western Substrate (FroggaBio) for chemiluminescence for 5 min. The CX37 primary antibody (Abcam, Cat# ab181701) was incubated overnight at 4°C at a 1:500 dilution. The same secondary antibody as used for CX43, anti-rabbit (Cell Signaling Technology, Cat# 7074), was used for CX37 at the same concentration as above for 1 h. The primary antibody for the loading control, GAPDH (Sigma-Aldrich, Cat# G8795), was incubated on the blot at a 1:25,000 dilution for 1 h. The secondary antibody, Anti-Mouse IgG, HRP-linked Antibody (Cell Signaling Technology, Cat# 7076) was used at a 1:5,000 dilution for 1 h. For each protein, the blot was imaged on a ChemiDoc XRS + Imaging System (Bio-Rad) to measure relative protein quantification. Six biological replicates were completed for CX37 and 3 biological replicates for CX43.

### TUNEL Staining of Blastocysts

Terminal deoxynucleotidyl transferase dUTP nick end labeling (TUNEL) staining was conducted on blastocysts to analyze the presence of DNA fragmentation. Fixed blastocysts were washed in PBS/PVA and placed in 0.5% Triton X in PBS for 60 min at room temperature. After 40 min, the positive control embryos were removed, washed twice in PBS/PVA and incubated in a solution of DNase I (Thermo Fisher Scientific) for the remaining 20 min in a dark, humidified chamber at 38.5°C. All embryos were then placed into the TUNEL reaction solution (1-part enzyme + 9-parts FITC label) and incubated for 60 min. The negative controls were exposed to FITC label only. All embryos then underwent two washes in 0.5% Triton X for 5 min followed by washes in PBS/PVA and RNase buffer and incubated with RNase A (Thermo Fisher) for 60 min in a dark, humidified chamber at 38.5°C. This was followed by two washes in RNase buffer and incubation in Hoechst stain for 30 min at 38.5°C. After the final incubation, blastocysts were prepared on slides and imaged using a Leica CTR5500B fluorescent microscope at the 20 × objective using the Open Lab software. Between 19 and 26 blastocysts were examined/group. Cell counting was completed manually, and trends verified using ImageJ software.

### Statistical Analysis

Statistical analysis was conducted using GraphPad Prism 6 and SPSS statistics software. In determining significance for rates, data were first tested for normality using Kolmogorov-Smirnov and Shapiro-Wilk tests, followed by a one-way analysis of variance (ANOVA) for those data sets that were normally distributed. For data that did not have a normal distribution, a Kruskal-Wallis test was carried out. For determining significance of proportions (i.e., spindle morphology), a chi-square test was utilized. Each experiment consisted of a minimum of 3 biological replicates and statistical significance was set at a two-tailed *p*-value < 0.05. Data sets determined to have this statistical significance then underwent Tukey's *post-hoc* test to compare the differences between treatment groups. Data shown represents the mean ± standard error of the mean (SEM).

## Results

### Oocyte Maturation Rates

**Figure 1:**
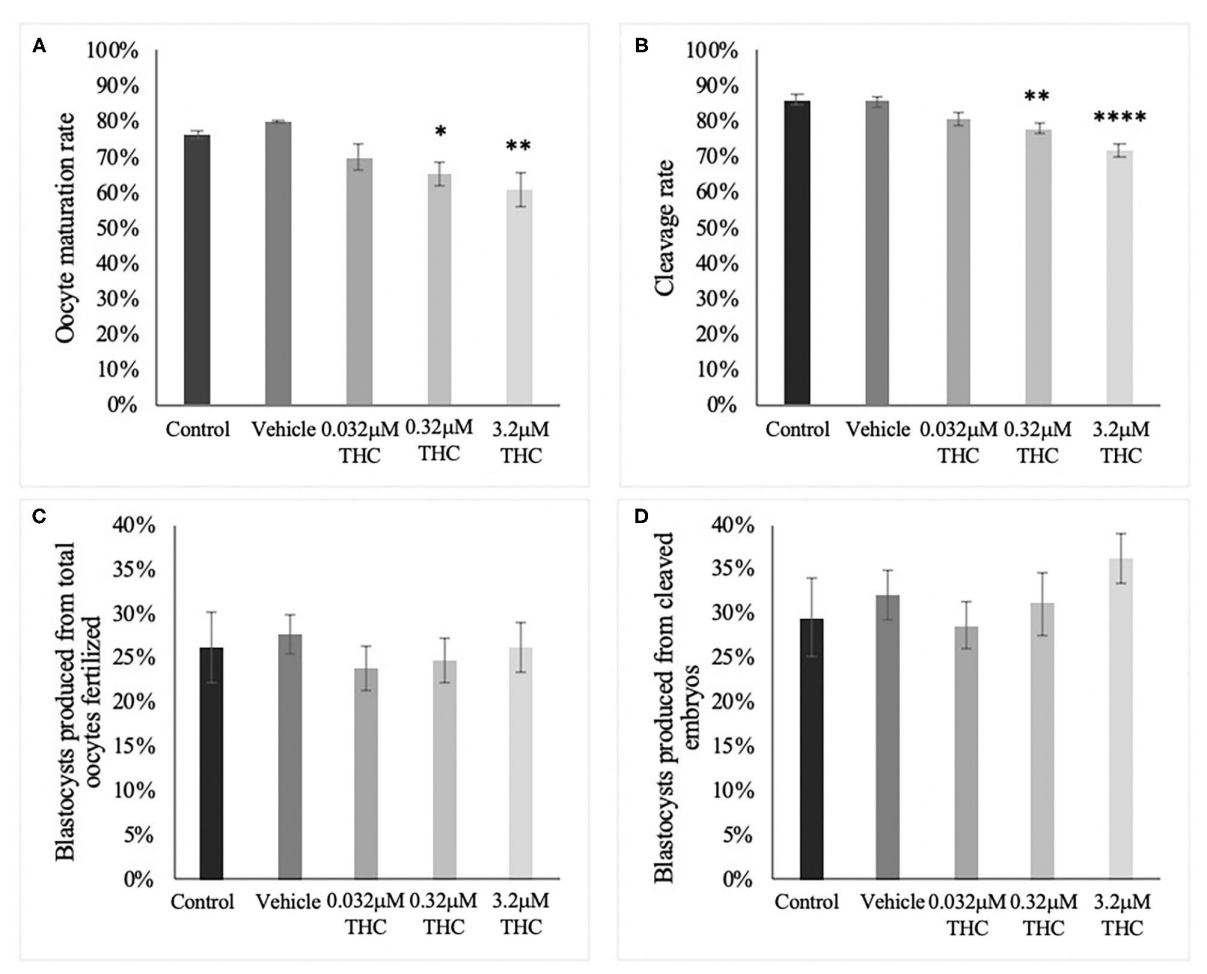
Developmental parameters after oocyte THC exposure. Oocytes were matured in varying doses of THC mimicking therapeutic cannabis use (low, 0.032 μM) or recreational cannabis use (mid, 0.32 μM; high, 3.2 μM), in addition to a control and vehicle (1:1:18 ethanol: tween: saline) groups. **(A)** Oocyte maturation rate. Oocytes were denuded from their cumulus cells after 24 h maturation and the presence of the first polar body was assessed as a determinant of a metaphase II state. The rate was calculated over the total number of oocytes. Three biological replicates of at least 50 oocytes/group was carried out, totalling 164 oocytes/group. **(B)** Cleavage rate. After 22 h maturation, oocytes were fertilized and cultured. The cleavage rate was assessed at 48 h post-fertilization and calculated over the number of total oocytes cultured. Eleven biological replicates consisting of at least 60 oocytes/group were analyzed for a total of 836 oocytes/group. **(C)** Blastocyst rate over the total number of oocytes. Embryos were cultured and rate of blastocyst development was measured at day 8 post-fertilization. Seven biological replicates of at least 60 oocytes/group were analyzed with a total of 507 oocytes/group used **(D)** Blastocyst rate calculated over cleavage rate. Bars represent mean ± SEM. Significance is calculated compared to vehicle group. *p < 0.05, **p < 0.01, ****p < 0.0001.

The percentage of oocytes with a visible polar body was assessed after 24 h of maturation in THC media (Figure 1A). The proportion of mature oocytes was comparable between the control and vehicle groups with an average rate of 76.46% (±0.01) and 80.12% (±0.004), respectively (*p* = 0.9126). Comparing the treatment groups with the vehicle showed a significant decrease in oocyte maturation with a 65.25% (±0.03) maturation rate in the mid (THC) dose (0.32 μM) (*p* = 0.0436) and a 60.85% (±0.05) maturation rate in the high ~~(THC) dose (3.2 μM) (*p* = 0.0094). The low (THC) group (0.032 μM) was also decreased, however not significantly (69.92% ± 0.04; *p* = 0.2159). Overall, these data display a negative dose-dependent correlation with increasing THC concentrations linked to reduced progression to the MII stage.

### Cleavage Rate

Percentage of embryos reaching the 2-cell stage by 48 h post-fertilization showed a similar trend as seen with oocyte maturation rates (Figure 1B). Firstly, there was no significant difference between the cleavage rate of the control (86.20% ± 0.02) and vehicle (85.53% ± 0.01) groups (*p* = 0.9979). As with the rates of oocyte maturation, the cleavage rates in the two doses representing recreational cannabis use were significantly decreased compared to the vehicle group with 78.06% (±0.01) cleavage rate in the mid (THC) (*p* = 0.0093) and 71.59% (±0.02) cleavage rate in the high (THC) group (*p* < 0.0001). There was a decrease in the low (THC) group, but this was again not significant (80.41% ± 0.02; *p* = 0.1558). These data display an overall negative dose-dependent correlation with increasing THC concentrations corresponding to decreased cleavage rate.

### Blastocyst Rate

Culture of THC-treated oocytes to the blastocyst stage led to no significant differences in the rate of blastocyst development in relation to the total number of oocytes as assessed at day 8 post-fertilization (*p* = 0.9217) (Figure 1C). These differences remained non-significant when calculated as blastocyst rate over the cleavage rate (*p* = 0.68) (Figure 1D).

### Spindle Morphology

**Figure 2:**
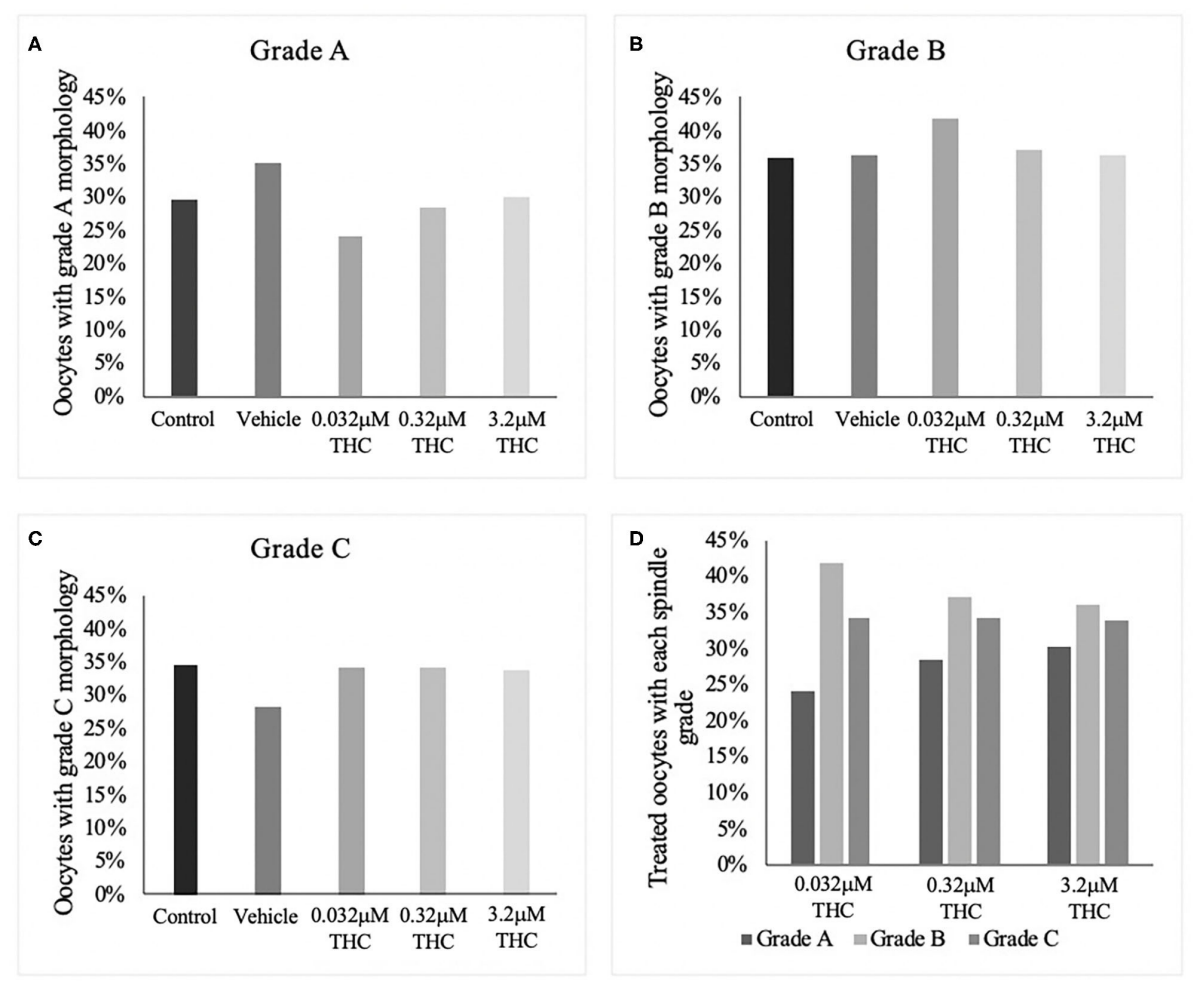
Spindle morphology assessment in metaphase II oocytes. **(A)** Oocytes with grade A spindles. **(B)** Oocytes with grade B spindles. **(C)** Oocytes with grade C spindles. **(D)** Proportion of each oocyte grade per treatment group. Oocytes were matured in media containing THC doses representing therapeutic cannabis use [low (THC) (0.032 μM)] and recreational cannabis use [mid (THC) (0.32 μM), high (THC) (3.2 μM)] in addition to a vehicle and control group for 24 h. Following denuding, oocytes displaying polar body extrusion were deemed at metaphase II and underwent immunocytochemistry staining for spindles. Spindle and chromosome morphology were graded and categorized, with grade A oocytes having chromosomes aligned on the metaphase plate and organized spindles. Grade C oocytes had misaligned chromosomes and disorganized spindles. Seventeen replicates of at least 5 oocytes/group were examined for a total of 70 and 83 oocytes/group. Bars represent the proportion of oocytes in each group.

In order to categorize morphological changes in spindle quality, oocytes underwent immunocytochemistry and were assessed into one of three arbitrary grading types: Grade A (chromosomal alignment, organized spindles), Grade B (slight chromosomal misalignment) or Grade C (chromosomes unaligned, disorganized spindles). A chi-square test identified no significant differences across treatment groups whether analyzed by group or by grade (Figures 2A–D). While not significant, the low (THC) group did have the lowest proportion of Grade A oocytes with 24.05% compared to 28.57 and 30.12% in the mid and high (THC) groups, respectively (*p* = 0.6801). Accordingly, the low (THC) group had the highest proportion of Grade B oocytes with 41.77%, compared to 37.14 and 36.14% in mid and high (*p* = 0.9363).

### Differential Staining

**Figure 3:**
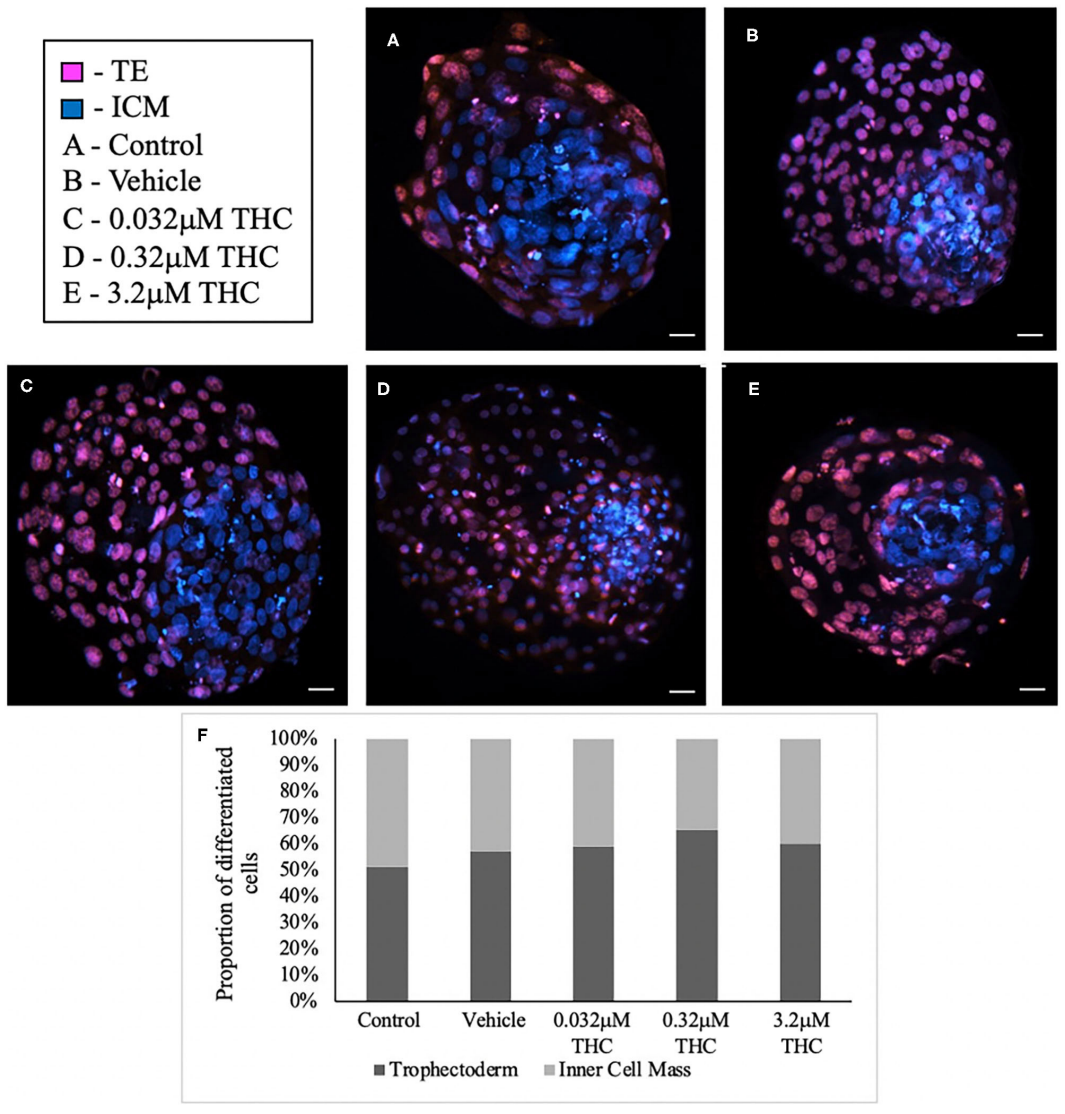
Differential staining of blastocysts produced from THC treated oocytes. **(A)** Control blastocyst. **(B)** Vehicle blastocyst. **(C)** Low (THC) (0.032 μM) blastocyst. **(D)** Mid (THC) (0.32 μM) blastocyst. **(E)** High (THC) (3.2 μM) blastocyst. **(F)** Proportion of trophectoderm (TE) vs. inner cell mass (ICM) cell numbers for each group. Oocytes underwent maturation in varying doses of THC representing therapeutic cannabis use [low (THC)] and recreational cannabis use [mid (THC), high (THC)] as well as vehicle or control, followed by fertilization and culture. Blastocysts were collected at 8 days post-fertilization and differentially stained. The trophectoderm (TE) was stained with propidium iodide (pink) and inner cell mass (ICM) with Hoechst stain (blue). Blastocysts were imaged with a Leica CTR5500B fluorescent microscope at the 20 × objective and cells manually counted. Each group had between 4 and 11 biological replicates (blastocysts). Bars represent the mean ± SEM. Scale bar =100 μm.

Staining blastocysts with propidium iodide followed by Hoechst led to differentiation between the inner cell mass (ICM), stained in blue, and trophectoderm (TE), stained in pink (Figures 3A–E). Counts of these cells revealed no significant difference in the proportion of ICM to TE between the treatment groups and the control/vehicle (ICM *p* = 0.6407; TE *p* = 0.4283) (Figure 3F). When comparing the proportions of cells attributed to the TE vs. ICM, there is a decreasing distribution of cells in the ICM with higher THC doses, however, this was not statistically significant. While the blastocyst rate was fairly similar among groups, only 4 blastocysts in the high (THC) group were capable of maintaining integrity during the experimental staining, suggesting the blastocysts treated with the highest THC doses might be more fragile.

### Nuclei Count

**Figure 4:**
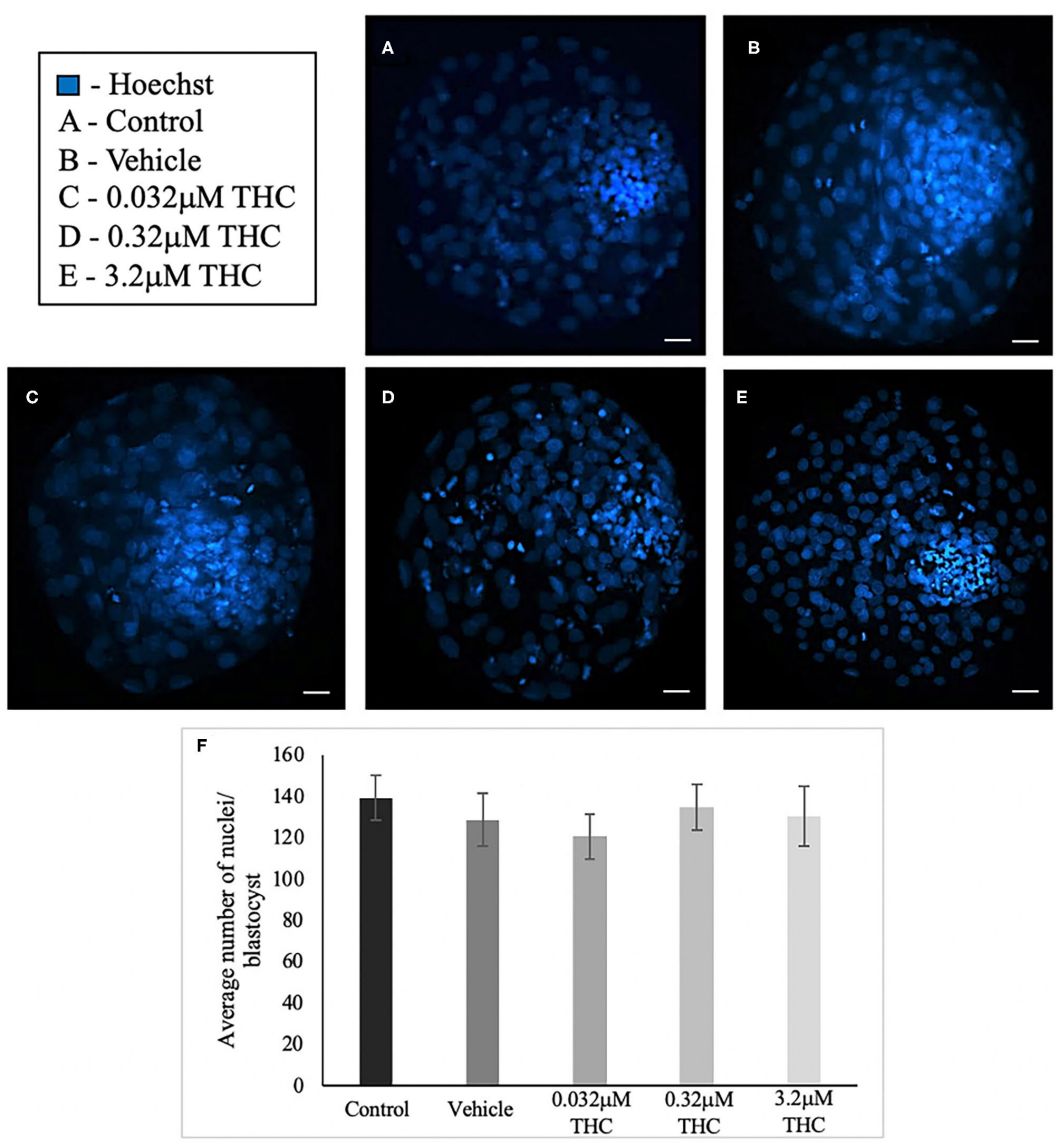
Total nuclei count in blastocysts produced from THC treated oocytes. **(A)** Control blastocyst. **(B)** Vehicle blastocyst. **(C)** Low (THC) (0.032 μM) blastocyst. **(D)** Mid (THC) (0.32 μM) blastocyst. **(E)** High (THC) (3.2 μM) blastocyst. **(F)** Total cell number. Oocytes were matured in doses of THC correlating to therapeutic cannabis use [low (THC)] and recreational cannabis use [mid (THC), high (THC)] as well as vehicle and control groups, followed by fertilization and culture to the blastocyst stage. Blastocysts were collected at 8 days post-fertilization and stained using either the differential staining or TUNEL protocol. Blastocysts were imaged with a Leica CTR5500B fluorescent microscope at the 20 × objective. Cells were counted manually and verified using ImageJ software. Each group had 25–34 biological replicates (blastocysts) analyzed. Bars represent the mean ± SEM. Scale bar =100 μm.

Blastocysts used for differential staining as well as TUNEL staining were analyzed to determine the total nuclei count. In total, between 25 and 34 blastocysts in each treatment were stained and nuclei counted (Figures 4A–E). Across the treatment groups there was no significant difference in the total cell numbers in blastocysts (*p* = 0.7694) (Figure 4F).

### Connexin mRNA Expression

**Figure 5:**
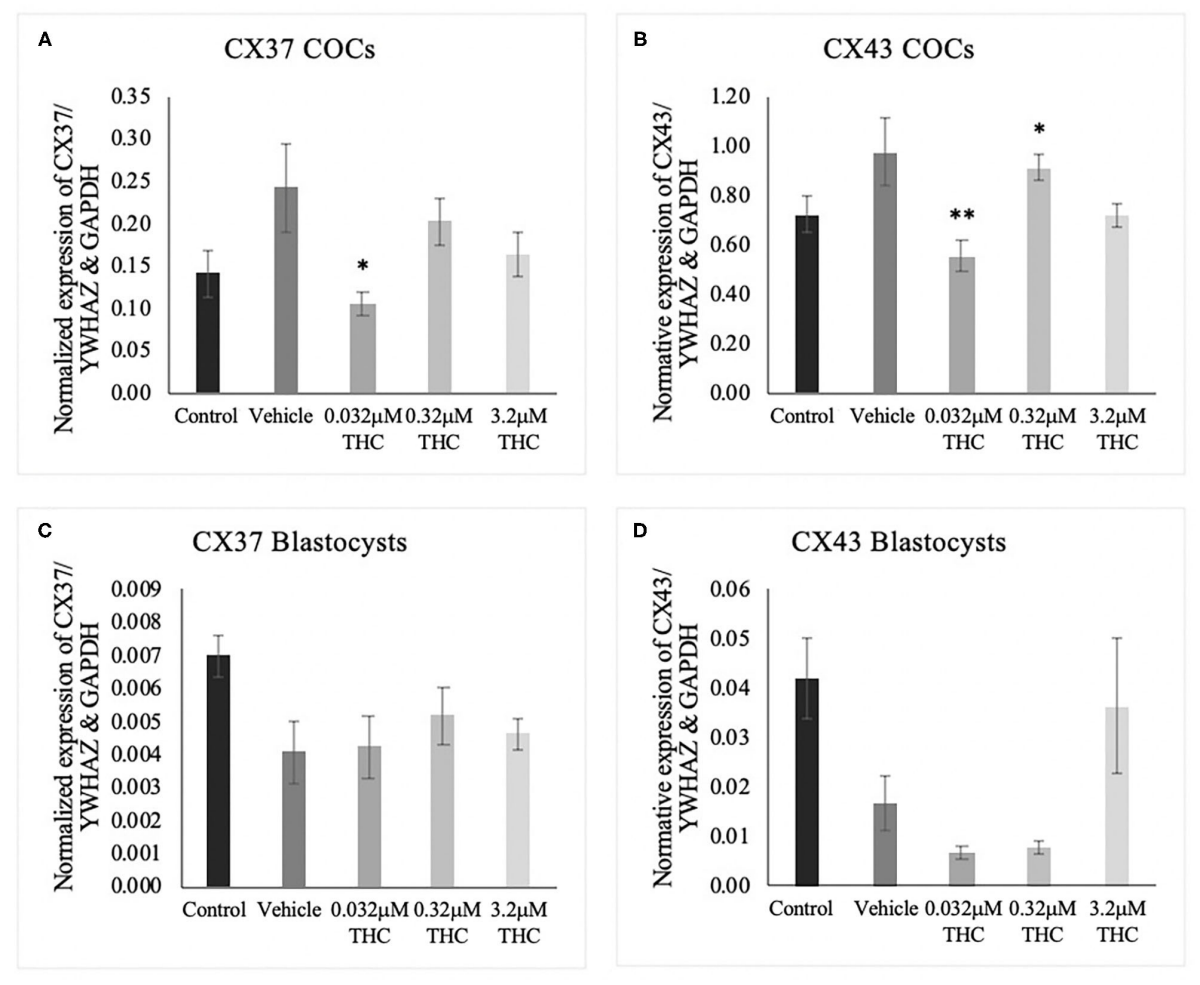
*Connexin* mRNA expression in cumulus-oocyte complexes (COCs) and blastocysts. **(A)** Normalized expression of *connexin 37* (CX37) in 250 ng of total mRNA from 15 COCs to reference genes YWHAZ and GAPDH. **(B)** Normalized *connexin 43* (CX43) expression in 250 ng of mRNA from 15 COCs. **(C)** Normalized expression of CX37 in 5 blastocysts. **(D)** Normalized expression of CX43 in 5 blastocysts. COCs were collected after 22 h of maturation in the treatment groups or fertilized, cultured and collected as blastocysts at day 8 post-fertilization. Total RNA was extracted and connexin mRNA was quantified using droplet digital PCR. Nine biological replicates were carried out in COCs and 5 biological replicates in blastocysts. Bars represent mean ± SEM. Significance is calculated in relation to the vehicle group. *p < 0.05, **p < 0.01.

cDNA from groups of 15 COCs and groups of 5 blastocysts were analyzed through ddPCR for expression of *connexin 37* (CX37) and *connexin 43* (CX43) transcripts. Connexin expression is highly correlated to oocyte quality and fertilization potential (Juneja et al., 1999; Jones, 2004; Houghton, 2005; Lodde et al., 2007; Luciano et al., 2011; Wang et al., 2012; Conti and Franciosi, 2018). In COCs, significant decreases in mRNA expression were identified between the vehicle and the low (THC) group of both *connexin 37* (*p* = 0.0377) and *connexin 43* (*p* = 0.0073) (Figures 5A,B). At the blastocyst level, there were no significant differences noted (CX37 *p* = 0.3535; CX43 *p* = 0.0948) (Figures 5C,D).

### Connexin Protein Expression

**Figure 6:**
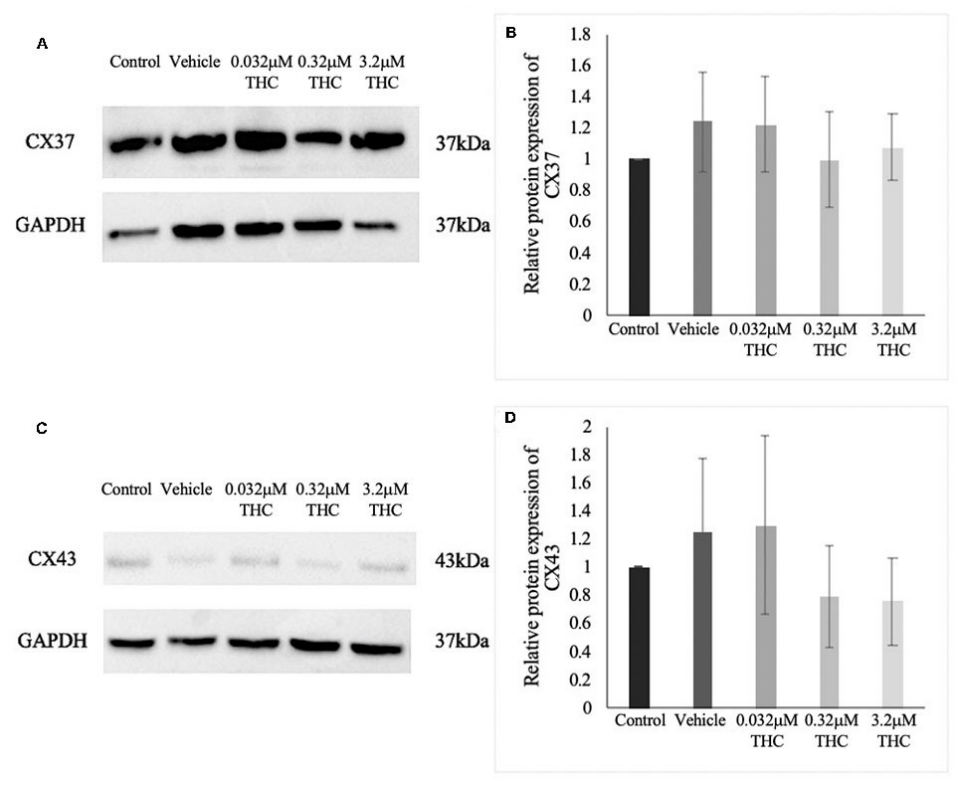
Expression of connexin protein in cumulus-oocyte complexes (COCs). **(A)** Western blot image of connexin 37 (CX37) and the corresponding GAPDH as the loading control. **(B)** Densitometric analysis of CX37 protein expression relative to GAPDH. **(C)** Western blot image of connexin 43 (CX43) and corresponding GAPDH. **(D)** Densitometric analysis of CX43 and relative GAPDH protein. Proteins were extracted from groups of 40 COCs after 22 h of maturation in one of the five treatment groups. Six biological replicates for CX37 and 3 biological replicates for CX43 were analyzed. Bars represent mean ± SEM.

Proteins were extracted from groups of 40 COCs and the levels of CX37 and CX43 were quantified relative to the loading control, GAPDH (Figures 6A–D). There were no significant differences noted in the expression of either CX37 or CX43 protein levels between the control and vehicle groups (*p* = 0.9628; *p* > 0.9999). Furthermore, the results for the treatment groups showed no significant differences in either CX37 or CX43 (*p* = 0.9316; *p* = 0.8253).

### TUNEL Staining of Blastocysts

**Figure 7:**
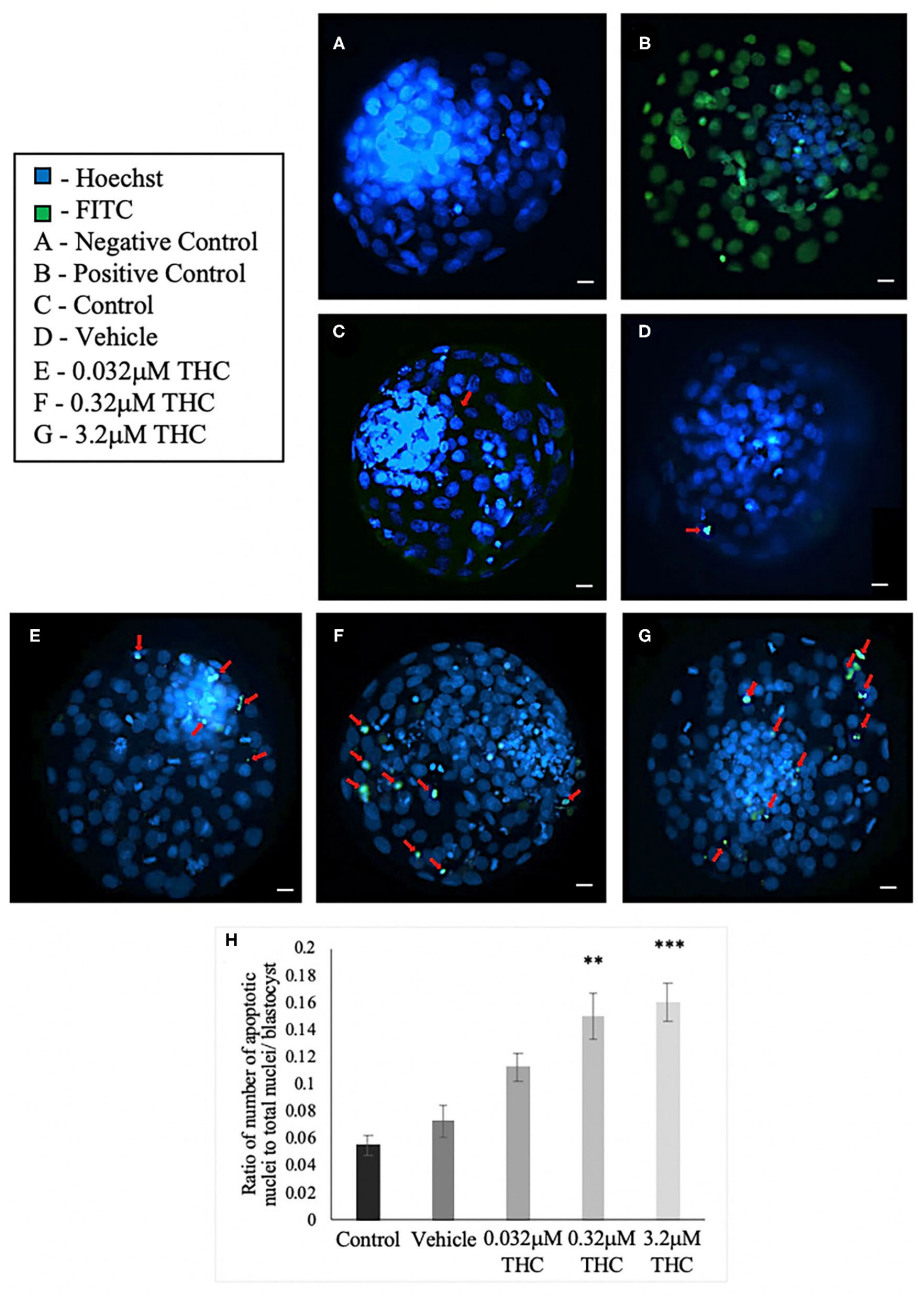
TUNEL staining of blastocysts. **(A)** Negative control blastocyst. **(B)** Positive control blastocyst (DNase I-treated). **(C)** Control blastocyst. **(D)** Vehicle blastocyst. **(E)** Low (THC) (0.032 μM) blastocyst. **(F)** Mid (THC) (0.32 μM) blastocyst. **(G)** High (THC) (3.2 μM) blastocyst. **(H)** Average DNA fragmentation over total number of nuclei. Cumulus-oocyte complexes (COCs) were matured in either THC, vehicle or control media followed by fertilization and culture for 8 days when blastocysts were collected. Blastocysts underwent TUNEL staining which stained nuclei undergoing DNA fragmentation with FITC label (green) and all nuclei with Hoechst stain (blue). Red arrows in **(C–G)** point out apoptotic cells. Blastocysts were imaged with a Leica CTR5500B fluorescent microscope at the 20 × objective. Between 19 and 26 total blastocysts per group were analyzed. Bars represent the mean ± SEM. Significance is calculated compared to vehicle group. Scale bar = 100 μm. **p < 0.01, ***p < 0.0001.

TUNEL staining was conducted to determine the level of apoptosis in blastocysts produced from THC-exposed oocytes (Figures 7A–G). There was no significant difference in the proportion of apoptotic cells between the control and vehicle groups (*p* > 0.9999). A dose-dependent effect, however, was identified with a significant increase in apoptotic cells in the mid (THC) (*p* = 0.0071) and high (THC) (*p* = 0.0006) groups, compared to the vehicle group (Figure 7H).

### Caspase-9 mRNA Expression

**Figure 8:**
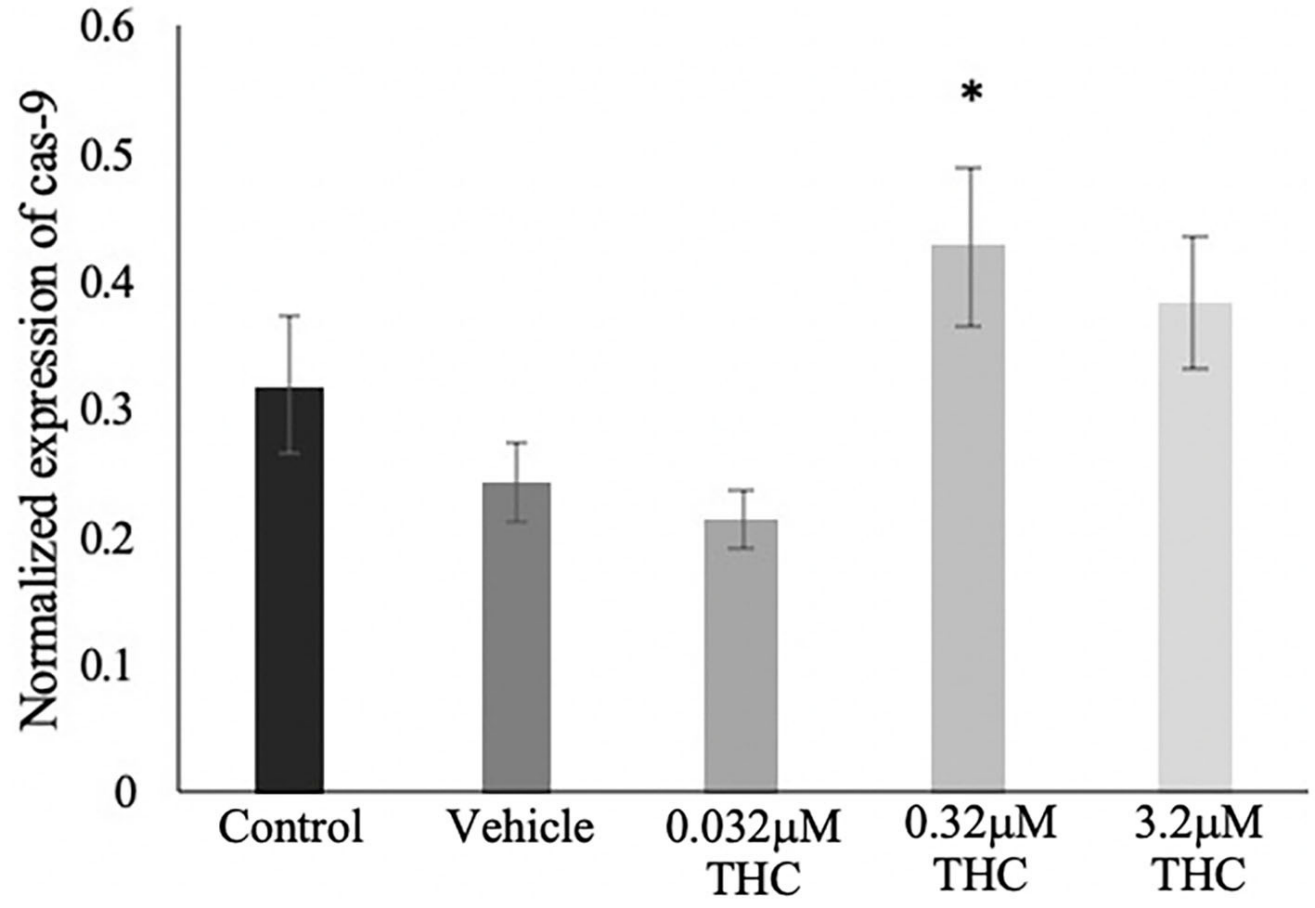
*Caspase 9* mRNA expression in blastocysts. Blastocysts were produced from cumulus-oocyte complexes (COCs) exposed to varying doses of THC representing therapeutic cannabis use (low, 0.032 μM) and recreational cannabis use (mid, 0.32 μM; high, 3.2 μM) in addition to a vehicle (1:1:18 ethanol: tween: saline) and control group for 22 h. COCs were fertilized and cultured to the blastocyst state. At day 8 post-fertilization, blastocysts were collected in groups of 5 and underwent RNA extraction followed by droplet digital PCR quantification of *caspase 9* (cas-9). Results were normalized to the reference gene, YWHAZ. Four biological replicates were analyzed. Bars represent mean ± SEM. Significance is calculated in relation to the vehicle group. *p < 0.05.

TUNEL results were validated using mRNA expression of *caspase-9* (cas-9), and showed a similar trend as the TUNEL staining (Figure 8). No significant difference was identified in the expression of *cas-9* between the control and vehicle groups (*p* = 0.7051), while increasing doses of THC displayed increasing expression of cas-9 mRNA levels. Only the mid (THC) exhibited statistical significance between the vehicle (*p* = 0.0408), with the high (THC) group trending toward significance at a *p*-value of 0.1604.

### CB1/CB2 Antagonists

**Figure 9:**
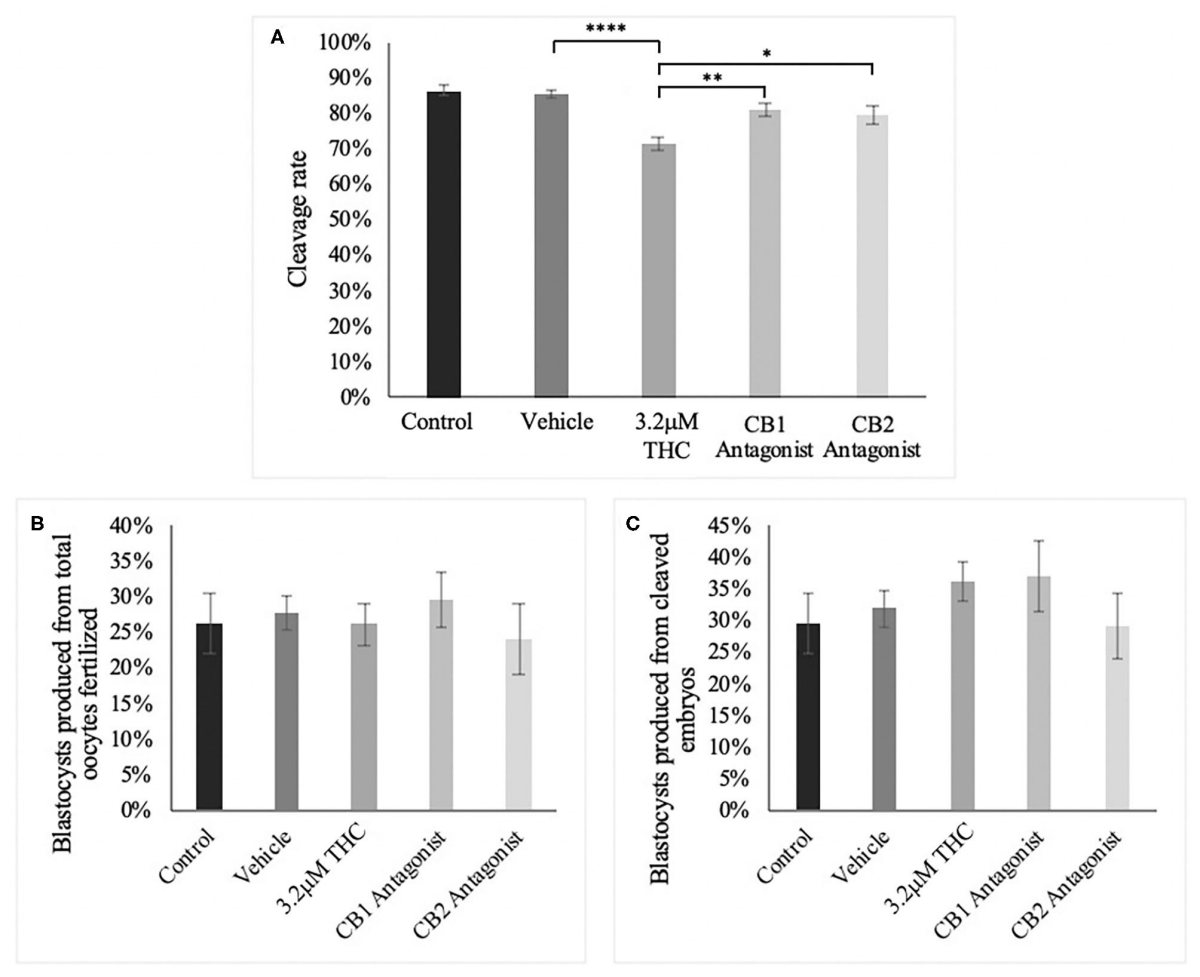
Developmental parameters of oocytes treated with THC and a selective cannabinoid antagonist. **(A)** Cleavage rate over total number of oocytes. Nine biological replicates of at least 60 oocytes/group were analyzed totalling 586 oocytes/group. **(B)** Blastocyst rate over total number of oocytes. Seven biological replicates of at least 60 oocytes/group was used for a total of 479 oocytes/group. **(C)** Blastocyst rate calculated over cleavage rate. Following the standard *in vitro* embryo production procedure, oocytes were matured in one of five groups: control, vehicle (1:1:18 ethanol:tween:saline), high (THC) (3.2 μM), CB1 antagonist (3.2 μM THC + 3.2 μM SR141716) and CB2 antagonist (3.2 μM THC + 3.2 μM SR144528). Mature cumulus oocyte complexes (COCs) were fertilized and cultured with the cleavage rate assessed at 48 h post-fertilization and blastocyst rate at day 8 post-fertilization. Bars represent the mean ± SEM. Significance is calculated compared to vehicle group. *p < 0.05, **p < 0.01, ****p < 0.0001.

Following the standard IVF procedure, oocytes were treated with the high (THC) dose in addition to either a CB1 or CB2 selective antagonist and developmental parameters assessed (Figure 9). Addition of a cannabinoid receptor antagonist resulted in normalization of cleavage rate back to levels comparable to the control groups (Figure 9A). This increase was significant between the high (THC) dose alone and with the addition of the CB1 antagonist (*p* = 0.0044) or with the addition of the CB2 antagonist (*p* = 0.0331). Addition of either antagonists resulted in no change to the percentage of day 8 blastocysts whether calculated over total oocyte number (*p* = 0.8841) or number of cleaved embryos at day two (*p* = 0.6488) (Figures 9B,C).

## Discussion

By treating oocytes *in vitro* with pharmacologically relevant doses of THC, [low (THC) 0.032 μM; mid (THC) 0.32 μM; high (THC) 3.2 μM], we attempted to model the effects of cannabis use on the female reproductive system, considering the lack of information on *in vivo* concentrations. Previous research has identified negative clinical outcomes associated with cannabis use, such as fewer oocytes retrieved and fewer embryos transferred in women undergoing IVF, disruption of ovulatory function and lower sperm concentration and sperm count in men (Mueller et al., 1990; Klonoff-Cohen et al., 2006; Gundersen et al., 2015). Our data add to this knowledge base by establishing preimplantation developmental changes following THC exposure.

These results demonstrate a significantly reduced ability of THC-treated oocytes to undergo nuclear maturation, and thereby resulting in a diminished ability to reach the 2-cell stage at the appropriate timepoint. Ultimately, this leads to decreased oocyte fertilization capability and poor early embryonic development, resulting in lower overall fertility. THC acts as a partial agonist at the CB1 and CB2 receptors, culminating in the activation of subsequent downstream signaling pathways (Maccarrone et al., 2010, 2015; Pertwee et al., 2010; Di Blasio et al., 2013; Walker et al., 2019). As inhibitory G-proteins, a known downstream effect is the inhibition of adenylyl cyclase resulting in cAMP loss and lowered activation of protein kinase A (PKA) (Howlett et al., 2002; Karasu et al., 2011; Cecconi et al., 2020). Moreover, this reduced PKA activity results in activation of maturation promoting factor (MPF) and further advancing the oocyte toward meiotic resumption (Jones, 2004; Norris et al., 2009; Adhikari and Liu, 2014; Conti and Franciosi, 2018).

Studies have suggested a role for the endocannabinoid system (ECS) in regulating these processes involved in oocyte maturation. Analysis of follicular fluid samples and their corresponding oocytes demonstrated a significant rise in AEA in mature follicles compared to their immature counterparts (Schuel et al., 2002; Lazzarin et al., 2004; El-Talatini et al., 2009). This suggests a role of the pathways regulated through eCB activation in coordinating the intricate processes that occur during oocyte maturation. Furthermore, studies using specific CB1 and CB2 antagonists have found a direct link between reduced cAMP loss and antagonism of the receptors, supporting a role of the CB receptors in regulating meiotic resumption via this pathway (Cecconi et al., 2019). Our study provides further evidence for this model.

The significant decrease in maturation and cleavage rate of oocytes exposed to the higher doses of THC allows us to speculate that THC might act as a selection factor for only the highest quality oocytes. As our study utilized a pool of oocytes, it is possible that the oocytes that were at a higher level of maturation prior to THC exposure were positively enhanced by the presence of THC in the maturation media. One possible speculation is that the addition of THC may have resulted in activation of the ECS and resumption of meiosis in developmentally competent oocytes, which mimics the natural rise in eCB concentration in follicular fluid that is correlated to oocyte maturity *in vivo* (El-Talatini et al., 2009). On the other hand, the more immature oocytes may have been negatively affected by THC by being pushed toward meiotic resumption prior to gaining developmental competence.

Interestingly, our results differ from those reported by López-Cardona and colleagues in 2016 in which they treated bovine oocytes with a 0.1 μM dose of either THC or a synthetic cannabinoid agonist, HU-210 and identified no differences in cleavage rate compared to the cont)rols (López-Cardona et al., 2016). This single concentration falls between our low (THC) (0.032 μM) and mid (THC) (0.32 μM) doses. Of particular note, however, is the differences in sample sizes, with our study analyzing 4.4 × more oocytes than the López-Cardona group (836 vs. 190 oocytes/group).

Both the López-Cardona study and ours, however, highlight a similar trend in the rates of blastocyst development with no significant difference identified (López-Cardona et al., 2016). This could again be due to only the highest quality oocytes being capable of maturation to the metaphase II stage prior to fertilization and thus possess the developmental capability to progress to the blastocyst stage.

Likewise, the concept of THC acting as a selection factor could also explain the not statistically significant changes in other parameters of quality assessment, including oocyte spindle morphology and embryo nuclei count. We again speculate that only the highest quality oocytes, which were positively impacted by the addition of THC, are able to properly resume meiosis with no impact on spindle morphology. The spindle morphology experiments only analyzed oocytes that had reached the metaphase II stage (selected by first polar body extrusion). Therefore, the lack of significant difference in spindle morphology in THC treated oocytes could be due to this initial sample selection. Thus, oocytes that are able to reach metaphase II under THC exposure displayed no changes to spindle morphology, however significantly reduced numbers of oocytes in the treatment groups were able to overcome this initial hurdle.

Similarly, those oocytes that were able to mature, fertilize and develop to the blastocyst stage displayed no differences in the number of nuclei whether assessed as a total number or differentiated into cells of the trophectoderm vs. the inner cell mass. This can again be linked to the initial effects of THC on oocyte maturation only allowing the highest quality oocytes to properly mature.

Another measurement of oocyte and embryo quality that was utilized in this study is the assessment of connexin mRNA and protein expression. Connexin expression has been found to be highly correlated to fertilization potential of oocytes and embryo quality (Juneja et al., 1999; Houghton, 2005; Lodde et al., 2007; Luciano et al., 2011; Wang et al., 2012). Our results demonstrated a significant decrease in the mRNA levels of two main connexins, *connexin 37* (CX37) and *connexin 43* (CX43), in oocytes and only in the low THC group. Interestingly the same results were not displayed in the higher THC doses. This may be due to the effects of CB1/CB2 mediated signaling molecules in the alteration of gene transcription coupled with the slower chromatin condensation and meiotic resumption in the lower THC group compared to its higher counterparts. Another potential avenue to consider is the effect of microRNAs which are known to bind to and degrade connexin transcripts (Salat-Canela et al., 2015). This could account for the lack of parallel findings between the mRNA levels and protein levels of connexins in oocytes, where no significant changes were identified at the protein level. Further research would need to be undertaken to explore this possible alteration of miRNAs by THC.

With the above changes attributed to ECS activation, the standard IVF protocol was repeated utilizing antagonists for the CB1 receptor (SR141716) and the CB2 receptor (SR144528) in addition to the highest THC dose. Supplementation with either antagonist resulted in a normalization of both cleavage and blastocyst rate compared to the high THC dose alone. These results further support the ability of THC to elicit its effects through CB receptor binding, as has been previously reported (Huestis et al., 1992; Pertwee et al., 2010; Walker et al., 2019). Research conducted by Cecconi and colleagues treated oocytes with only the cannabinoid receptor antagonists and noted a significant decrease in the rate of intracellular cAMP loss compared to controls (Cecconi et al., 2019). This outcome again strengthens our results by demonstrating the role the ECS plays in controlling cAMP levels and thereby regulating meiotic resumption.

Furthermore, a well-described downstream effect of ECS activation is the induction of a pro-apoptotic state (Fonseca et al., 2013; De Domenico et al., 2017; Cecconi et al., 2020). This was evaluated and characterized in our study using blastocysts produced from THC-exposed oocytes. TUNEL staining in combination with quantification of *caspase-9* mRNA expression demonstrated a significant increase in apoptotic cells in both the mid and high THC groups. These results support the mechanistic action of THC to promote apoptosis via hyperactivation of the ECS. Likewise, studies using human cytotrophoblast cells have found similar increases in caspase expression when exposed to a biosynthetic form of the endogenous cannabinoid, 2-AG (Costa et al., 2014). The addition of 2-AG also resulted in loss of mitochondrial membrane potential and an increase in reactive oxygen species (ROS) which suggests involvement of the mitochondria as a direct effect of ECS activation (Costa et al., 2014). Our results support this link between the ECS and apoptosis and the ability of THC to mimic the eCBs and activate these pathways.

In conclusion, this study provides evidence for the negative effects of THC on the female reproductive system, and specifically on the intricate processes involved in oocyte maturation and early preimplantation development. This is in line with previous research aimed to identify clinical outcomes associated with cannabis use that showed poorer oocyte quality and reduced rates of embryo transfer (Mueller et al., 1990; Lazzarin et al., 2004; Klonoff-Cohen et al., 2006). Those oocytes that are capable of overcoming this initial stressor and are capable of developing to the blastocyst level display significant increases in levels of apoptosis compared to controls. This results in diminished ability of the embryos to successfully implant and produce a viable clinical pregnancy.

## Data Availability Statement

The original contributions presented in the study are included in the article/supplementary material, further inquiries can be directed to the corresponding author.

## Author Contributions

MM significantly contributed to the study design, manuscript writing, and performed the majority of the experiments and analysis. AT, JD, and LS performed some experiments and analysis. JK participated in the study design. LF contributed to the scientific idea of the manuscript, to the study design drafting the experiments and considerably participated in the manuscript writing. All authors contributed to the article and approved the submitted version.

## Conflict of Interest

The authors declare that the research was conducted in the absence of any commercial or financial relationships that could be construed as a potential conflict of interest.

## References

[B1] Adhikari D. Liu K. (2014). The regulation of maturation promoting factor during prophase I arrest and meiotic entry in mammalian oocytes. Mol. Cell Endocrinol. 382, 480–487. 10.1016/j.mce.2013.07.027 23916417

[B2] Amoako A. A. Marczylo T. H. Marczylo E. L. Elson J. Willets J. M. Taylor A. H.. (2013). Anandamide modulates human sperm motility: implications for med with asthenozoospermia and oligoasthenoteratozoospermia. Hum. Reprod. 28, 2058–2066. 10.1093/humrep/det232 23697839

[B3] Anderson K. Nisenblat V. Norman R. (2010). Lifestyle factors in people seeking infertility treatment-a review. Aust. N. Z. J. Obstet. Gynaecol. 50, 8–20. 10.1111/j.1479-828X.2009.01119.x 20218991

[B4] Battista N. Pasquariello N. Di Tommaso M. Maccarrone M. (2008). Interplay between endocannabinoids, steroids and cytokines in the control of human reproduction. J. Neuroendocrinol. 20, 82–89. 10.1111/j.1365-2826.2008.01684.x 18426505

[B5] Bustin S. A. Benes V. Garson J. A. Hellemans J. Huggett J. Kubista M.. (2009). The MIQE guidelines: minimum information for publication of quantitative real-time PCR experiments. Clin. Chem. 55, 611–622. 10.1373/clinchem.2008.112797 19246619

[B6] Campbell B. K. Souza C. Gong J. Webb R. Kendall N. Marsters P.. (2003). Domestic ruminants as models for the elucidation of the mechanisms controlling ovarian development in humans. Reprod. Suppl. 61, 429–443. 10.1530/biosciprocs.5.032 14635953

[B7] Cecconi S. Rapino C. Di Nisio V. Rossi G. Maccarrone M. (2020). The (endo)cannabinoid signalling in female reproduction: what are the latest advances? Prog. Lipid. Res. 77:101019. 10.1016/j.plipres.2019.101019 31862482

[B8] Cecconi S. Rossi G. Oddi S. Di Nisio V. Maccarrone M. (2019). Role of major endocannabinoid binding receptors during mouse oocyte maturation. Int. J. Mol. Sci. 20:2866. 10.3390/ijms20122866 31212770 PMC6627642

[B9] Chavarro J. E. (2018). Marijuana and reproduction: time to raise the evidence bar to a new high. Fertil. Steril. 109, 793–794. 10.1016/j.fertnstert.2018.01.045 29778376

[B10] Childers S. R. Breivogel C. S. (1998). Cannabis and endogenous cannabinoid systems. Drug Alcohol. Depend. 51, 173–187. 10.1016/S0376-8716(98)00075-1 9716939

[B11] Conti M. Franciosi F. (2018). Acquisition of oocyte competence to develop as an embryo: integrated nuclear and cytoplasmic events. Hum. Reprod. Update 24, 245–266. 10.1093/humupd/dmx040 29432538 PMC5907346

[B12] Correa F. Wolfson M. L. Valchi P. Aisemgerg J. Franchi A. M. (2016). Endocannabinoid system and pregnancy. Reproduction 152, R191–R200. 10.1530/REP-16-0167 27798285

[B13] Corsi D. J. Hsu H. Weiss D. Fell D. B. Walker M. (2019). Trends and correlates of cannabis use in pregnancy: a population-based study in Ontario, Canada from 2012 to 2017. Can. J. Publ. Health 110, 76–84. 10.17269/s41997-018-0148-0 30387034 PMC6335373

[B14] Costa M. A. Fonseca B. M. Keating E. Teixera N. A. Correia-da-Silva G. (2014). 2- arachidonoylglycerol effects in cytotrophoblasts: metabolic enzymes expression and apoptosis in BeWo cells. Reproduction 147, 301–311. 10.1530/REP-13-0563 24324206

[B15] Dalton G. D. Bass C. E. Van Horn C. Howlett A. C. (2009). Signal transduction via cannabinoid receptors. CNS Neurol. Disord. Drug Targets 8, 422–431. 10.2174/187152709789824615 19839935 PMC3976677

[B16] De Angelis C. Nardone A. Garifalos F. Pivonello C. Sansone A. Conforti A.. (2020). Smoke, alcohol and drug addiction and female fertility. Reprod. Biol. Endocrinol. 18:21. 10.1186/s12958-020-0567-7 32164734 PMC7069005

[B17] De Domenico E. Todaro F. Rossi G. Dolci S. Geremia R. Rossi P.. (2017). Overactive type 2 cannabinoid receptor induces meiosis in fetal gonads and impairs ovarian reserve. Cell Death Dis. 8:e3085. 10.1038/cddis.2017.496 28981118 PMC5682662

[B18] De Felici M. Klinger F. G. Farini D. Scaldaferri M. L. Iona S. Lobascio M. (2005). Establishment of oocyte population in the fetal ovary: primordial germ cell proliferation and oocyte programmed cell death. Reprod. Biomed. Online 10, 182–191. 10.1016/S1472-6483(10)60939-X 15823221

[B19] Di Blasio A. M. Vignali M. Gentillini D. (2013). The endocannabinoid pathways and the female reproductive organs. J. Mol. Endocrinol. 50, R1–R9. 10.1530/JME-12-0182 23178290

[B20] El Sohly M. A. Mehmedic Z. Foster S. Gon C. Chandra S. Church J. C. (2016). Changes in cannabis potency over the last two decades (1995-2014): analysis of current data in the United States. Biol. Psychiatry 79, 613–619. 10.1016/j.biopsych.2016.01.004 26903403 PMC4987131

[B21] El-Talatini M. R. Taylor A. H. Elson J. C. Brown L. Davidson A. C. Konje J. C. (2009). Localization and function of the endocannabinoid system in the human ovary. PloS ONE 4:e4579. 10.1371/journal.pone.0004579 19238202 PMC2640464

[B22] FDA (2004). Marinol© (Dronabinol) Capsules. Available online at: https://www.accessdata.fda.gov/drugsatfda_docs/label/2005/018651s021lbl.pdf (accessed July 20, 2020).

[B23] Ferris J. Mahboubi K. MacLusky N. King W. A. Favetta L. A. (2016). BPA exposure during *in vitro* oocyte maturation results in dose-dependent alterations to embryo development rates, apoptosis rate, sex ratio and gene expression. Reprod. Toxicol. 59, 129–138. 10.1016/j.reprotox.2015.12.002 26686065

[B24] Fonseca B. M. Correia-da-Silva G. Teixeira N. A. (2013). The endocannabinoid anandamide induces apoptosis of rat decidual cells through a mechanism involving ceramide synthesis and p38 MAPK activation. Apoptosis 18, 1526–1535. 10.1007/s10495-013-0892-9 24048885

[B25] Guindon J. Hohmann A. G. (2011). The endocannabinoid system and cancer: therapeutic implication. Br. J. Pharmacol. 163, 1447–1463. 10.1111/j.1476-5381.2011.01327.x 21410463 PMC3165955

[B26] Gundersen T. D. Jørgensen N. Andersson A. M. Bang A. K. Nordkap L. Skakkebæk N. E.. (2015). Association between use of marijuana and male reproductive hormones and semen quality: a study among 1,215 healthy young men. Am. J. Epidemiol. 182, 473–481. 10.1093/aje/kwv135 26283092

[B27] Houghton F. D. (2005). Role of gap junctions during early embryo development. Reproduction 129, 129–135. 10.1530/rep.1.00277 15695607

[B28] Howlett A. C. Barth F. Bonner T. I. Cabral G. Casellas P. Devane W. A.. (2002). International union of pharmacology. XXVII. Classification of cannabinoid receptors. Pharmacol. Rev. 54, 161–202. 10.1124/pr.54.2.161 12037135

[B29] Huestis M. A. Sampson A. H. Holicky B. J. Henningfield J. E. Cone E. J. (1992). Characterization of the absorption phase of marijuana smoking. Clin. Pharmacol. Ther. 52, 31–41. 10.1038/clpt.1992.100 1320536

[B30] Ilnitsky S. Van Uum S. (2019). Marijuana and fertility. CMAJ 191:E638. 10.1503/cmaj.181577 31182459 PMC6565391

[B31] Jones K. T. (2004). Turning it on and off: M-phase promoting factor during meiotic maturation and fertilization. Mol. Hum. Reprod. 10, 1–5. 10.1093/molehr/gah009 14665700

[B32] Joshi N. Onaivi E. S. (2019). Endocannabinoid system components: overview and tissue distribution, in Recent Advances in Cannabinoid Physiology and Pathology, ed Bukiya A. (Cham: Springer), 1–12. 10.1007/978-3-030-21737-2_1 31332731

[B33] Juneja S. C. Barr K. J. Enders G. C. Kidder G. M. (1999). Defects in the germline and gonads of mice lacking connexin 43. Biol. Reprod. 60, 1263–1270. 10.1095/biolreprod60.5.1263 10208994

[B34] Karasu T. Marczylo T. H. Maccarrone M. Konje J. C. (2011). The role of sex steroid hormones, cytokines and the endocannabinoid system in female fertility. Hum. Reprod. Update 17, 347–361. 10.1093/humupd/dmq058 21227997

[B35] Klonoff-Cohen H. S. Natarajan L. Chen R. V. (2006). A prospective study of the effects of female and male marijuana use on *in vitro* fertilization (IVF) and gamete intrafallopian transfer (GIFT) outcomes. Am. J. Obstet. Gynecol. 194, 369–376. 10.1016/j.ajog.2005.08.020 16458631

[B36] Lazzarin N. Valensise H. Bari M. Ubaldi F. Battista N. Finazzi-Agrò A.. (2004). Fluctuations of fatty acid amide hydrolase and anandamide levels during the human ovulatory cycle. Gynecol. Endocrinol. 18, 212–218. 10.1080/09513590410001692492 15293893

[B37] Lemberger L. Axelrod J. Kopin I. J. (1971). Metabolism and distribution of tetrahydrocannabinols in naïve subjects and chronic marijuana users. Ann. N.Y. Acad. Sci. 191, 142–154. 10.1111/j.1749-6632.1971.tb13994.x

[B38] Lodde V. Modina S. Galbusera C. Franciosi F. Luciano A. M. (2007). Large-scale chromatin remodeling in germinal vesicle bovine oocytes: interplay with gap junction functionality and developmental competence. Mol. Reprod. Dev. 74, 740–749. 10.1002/mrd.20639 17075796

[B39] López-Cardona A. P. Sánchez-Calabuig M. J. Beltran-Breña P. Agirregoitia N. Rizos D. Agirregoitia E.. (2016). Exocannabinoids effect on *in vitro* bovine oocyte maturation via activation of AKT and ERK1/2. Reproduction 152, 603–612. 10.1530/REP-16-0199 27798282

[B40] Luciano A. M. Franciosi F. Lodde V. Corbani D. Lazzari G. Crotti G.. (2010). Transferability and inter-laboratory variability assessment of the *in vitro* bovine oocyte maturation (IVM) test within ReProTect. Reprod. Toxicol. 30, 81–88. 10.1016/j.reprotox.2010.01.015 20156549

[B41] Luciano A. M. Franciosi F. Modina S. C. Lodde V. (2011). Gap junction-mediated communications regulate chromatin remodelling during bovine oocyte growth and differentiation through cAMP-dependent mechanisms. Biol. Reprod. 85, 1252–1259. 10.1095/biolreprod.111.092858 21816847

[B42] Maccarrone M. Bab I. Bíró T. Cabral G. A. Dey S. K. Di Marzo V.. (2015). Endocannabinoid signalling at the periphery: 50 years after THC. Trends. Pharmacol. Sci. 36, 277–296. 10.1016/j.tips.2015.02.008 25796370 PMC4420685

[B43] Maccarrone M. Dainese E. Oddi S. (2010). Intracellular trafficking of anandamide: new concepts for signalling. Trends Biochem. Sci. 35, 601–608. 10.1016/j.tibs.2010.05.008 20570522

[B44] Maykut M. O. (1985). Health consequences of acute and chronic marihuana use. Prog. Neuropsychopharmacol. Biol. Psychiatry. 9, 209–238. 10.1016/0278-5846(85)90085-5 3898227

[B45] Mechoulam R. Gaoni Y. (1965). Hashish—IV: the isolation and structure of cannabinolic cannabidiolic and cannabigerolic acids. Tetrahedron 21, 1223–1229. 10.1016/0040-4020(65)80064-3 5879350

[B46] Ménezo Y. J. R. Hérubel F. (2002). Mouse and bovine models for human IVF. Reprod. Biomed. Online 4, 170–175. 10.1016/S1472-6483(10)61936-0 12470581

[B47] Mueller B. A. Baling J. R. Weiss N. S. Moore D. E. (1990). Recreational drug use and the risk of primary infertility. Epidemiology 1, 195–200. 10.1097/00001648-199005000-00003 2081252

[B48] National Institute on Drug Abuse (2018). National Survey on Drug Use and Health: Trends in Prevalence of Various Drugs for Ages 12 or Older, Ages 12 to 17, Ages 18 to 25, and Ages 26 or Older; 2015 - 2017 (in percent). Available online at: https://www.drugabuse.gov/drug-topics/trends-statistics/national-drug-early-warning-system-ndews/national-survey-drug-use-health (accessed July 20, 2020).

[B49] Norris R. P. Ratzan W. J. Freudzon M. Mehlmann L. M. Krall J. Movsesian M. A.. (2009). Cyclic GMP from the surrounding somatic cells regulates cyclic AMP and meiosis in the mouse oocyte. Development 136, 1869–1878. 10.1242/dev.035238 19429786 PMC2680110

[B50] Paria B. C. Song H. Wang X. Schmid P. C. Krebsbach R. J. Schmid H. H.. (2001). Dysregulated cannabinoid signaling disrupts uterine receptivity for embryo implantation. J. Biol. Chem. 276, 20523–20528. 10.1074/jbc.M100679200 11279117

[B51] Park B. McPartland J. M. Glass M. (2004). Cannabis, cannabinoids and reproduction. Prostaglandins Leukot Essent Fatty Acids 70, 189–197. 10.1016/j.plefa.2003.04.007 14683692

[B52] Pertwee R. G. Howlett A. C. Abood M. E. Alexander S. P. H. Di Marzo V. Elphick M. R.. (2010). International union of basic and clinical pharmacology. LXXIX. Cannabinoid receptors and their ligands: beyond CB_1_ and CB_2_. Pharmacol. Rev. 62, 588–631. 10.1124/pr.110.003004 21079038 PMC2993256

[B53] Salat-Canela C. Muñoz M. J. Sesé M. Ramón y Caial S. Aasen T. (2015). Post-transcriptional regulation of connexins. Biochem. Soc. Trans. 43, 465–470. 10.1042/BST20150033 26009192

[B54] Sánchez F. Smitz J. (2012). Molecular control of oogenesis. Biochim. Biophys. Acta. 1822, 1896–1912. 10.1016/j.bbadis.2012.05.013 22634430

[B55] Santos R. R. Schoevers E. J. Roelen B. A. (2014). Usefulness of bovine and porcine IVM/IVF models for reproductive toxicology. Reprod. Biol. Endocrinol. 12:117. 10.1186/1477-7827-12-117 25427762 PMC4258035

[B56] Schmid P. C. Paria B. C. Krebsbach R. J. Schmid H. H. O. Dey S. K. (1997). Changes in anandamide levels in mouse uterus are associated with uterine receptivity for embryo implantation. Proc. Natl. Acad. Sci. U.S.A. 94, 4188–4192. 10.1073/pnas.94.8.4188 9108127 PMC20598

[B57] Schuel H. Burkman L. J. Lippes J. Crickard K. Forester E. Piomelli D.. (2002). N-Acylethanolamines in human reproductive fluids. Chem. Phys. Lipids 121, 211–227. 10.1016/S0009-3084(02)00158-5 12505702

[B58] Sharma J. (2016). Role of hippo signaling pathway in bovine preimplantation embryo development (dissertation). University of Guelph, Guelph, ON, Canada.

[B59] Statistics Canada (2020). Table 13-10-0383-01, Prevalence of cannabis use in the past three months, self-reported.

[B60] Taylor A. H. Ang C. Bell S. C. Konje J. C. (2007). The role of the endocannabinoid system in gametogenesis, implantation and early pregnancy. Hum. Reprod. Update 13, 501–513. 10.1093/humupd/dmm018 17584820

[B61] Tesfaye D. Lonergan P. Hoelker M. Rings F. Nganvongpanit K. Havlicek V.. (2007). Suppression of connexin 43 and E-cadherin transcripts in *in vitro* derived bovine embryos following culture *in vitro* or *in vivo* in the homologous bovine oviduct. Mol. Reprod. Dev. 74, 978–988. 10.1002/mrd.20678 17219420

[B62] Tscherner A. (2017). MicroRNA expression and regulation in the cumulus-oocyte complex and preimplantation embryo (dissertation). University of Guelph, Guelph, ON, Canada.

[B63] Van Heertum K. Rossi B. (2017). Alcohol and fertility: how much is too much? Fertil. Res. Pract. 3:10. 10.1186/s40738-017-0037-x 28702207 PMC5504800

[B64] van Woudenberg BA. Gröllers-Mulderii M. Snel C. Jeurissen N. Stierum R. Wolterbeek A. (2012). The bovine oocyte *in vitro* maturation model: a potential tool for reproductive toxicology screening. Reprod. Toxicol. 34, 251–260. 10.1016/j.reprotox.2012.05.098 22664270

[B65] Walker O. Holloway A. C. Raha S. (2019). The role of the endocannabinoid system in female reproductive tissues. J. Ovarian Res. 12:3. 10.1186/s13048-018-0478-9 30646937 PMC6332911

[B66] Wang H. Xie H. Dey S. K. (2006). Endocannabinoid signalling directs periimplantation events. AAPS J. 8, E425–E432. 10.1007/BF02854916 16808046 PMC3231559

[B67] Wang Q. Chi M. M. Schedl T. Moley K. H. (2012). An intracellular pathway for glucose transport into mouse oocytes. Am. J. Physiol. Endocrinol. Metab. 302, E1511–E1518. 10.1152/ajpendo.00016.2012 22473000 PMC3378161

[B68] Westfall R. E. Janssen P. A. Lucas P. Capler R. (2006). Survey of medicinal cannabisuse among childbearing women: patters of its use in pregnancy and retroactive self-assessment of its efficacy against “morning sickness”. Complement. Ther. Clin. Pract. 12, 27–33. 10.1016/j.ctcp.2005.09.006 16401527

[B69] Whan L. B. West M. S. L. McClure N. Lewis S. E. M. (2006). Effects of delta-9-tetrahydrocannabinol, the primary psychoactive cannabinoid in marijuana, on human sperm function *in vitro*. Fertil. Steril. 85, 653–660. 10.1016/j.fertnstert.2005.08.027 16500334

[B70] Yuan X. Chen Z. Yang Z. Gao J. Zhang A. Wu S. M.. (2008). Expression pattern of connexins in the corneal and limbal epithelium of a primate. Cornea 28, 194–199. 10.1097/ICO.0b013e318185268e 19158564

